# Precise Measurement of the Stoichiometry of the Adaptive Bacterial Flagellar Switch

**DOI:** 10.1128/mbio.00189-23

**Published:** 2023-03-22

**Authors:** Antai Tao, Guangzhe Liu, Rongjing Zhang, Junhua Yuan

**Affiliations:** a Department of Physics, University of Science and Technology of China, Hefei, Anhui, China; b Wenzhou Institute, University of Chinese Academy of Science, Wenzhou, Zhejiang, P.R. China; c School of Engineering and Science, University of Chinese Academy of Science, Beijing, P.R. China; University of Utah

**Keywords:** adaptive remodeling, allostery, bacterial motility, flagellar motor

## Abstract

The cytoplasmic ring (C-ring) of the bacterial flagellar motor controls the motor rotation direction, thereby controlling bacterial run-and-tumble behavior. The C-ring has been shown to undergo adaptive remodeling in response to changes in motor directional bias. However, the stoichiometry and arrangement of the C-ring is still unclear due to contradiction between the results from fluorescence studies and cryo-electron microscopy (cryo-EM) structural analysis. Here, by using the copy number of FliG molecules (34) in the C-ring as a reference, we precisely measured the copy numbers of FliM molecules in motors rotating exclusively counterclockwise (CCW) and clockwise (CW). We surprisingly found that there are on average 45 and 58 FliM molecules in CW and CCW rotating motors, respectively, which are much higher than previous estimates. Our results suggested a new mechanism of C-ring adaptation, that is, extra FliM molecules could be bound to the primary C-ring with probability depending on the motor rotational direction. We further confirmed that all of the FliM molecules in the C-ring function in chemotaxis signaling transduction because all of them could be bound by the chemotactic response regulator CheY-P. Our measurements provided new insights into the structure and arrangement of the flagellar switch.

## INTRODUCTION

Flagellated bacteria swim by rotating the flagellum (1), which is composed of the following three parts: the rotary motor acting as the basal body, the filament acting as the helical propeller, and the hook acting as the universal joint that connects the rotary motor and the filament. The flagellar motor is embedded in the cell envelope to drive the rotation of the flagellum. The Gram-negative bacteria Escherichia coli and Salmonella enterica serovar Typhimurium (*Salmonella* for short) are the best-studied model organisms that provide detailed insights into the structure, assembly, and function of the flagellar motor (2). In these two bacterial species, the core of the motor, the rotor, is composed of C-ring, MS-ring, LP-ring, and the rod and is surrounded by a ring of stator units. The C-ring, also called the switch complex, is localized to the cytoplasmic region of the rotor and is composed of three types of proteins as follows: FliG, FliM, and FliN (1). The C-ring is one of the conserved features, although structural diversity has been shown in different bacterial species (3). Typically, Helicobacter pylori, Bacillus subtilis, and Thermotoga maritima contain FliY as a supplementary subunit to or instead of FliN (4). The core set of components of the bacterial flagellar motor is highly conserved among bacterial species (5), although some structures are broadly but not universally distributed across flagellated bacterial species; for example, motors of firmicutes lack the LP-ring, probably associated with the lack of the outer membrane in these bacteria (6). In *Vibrio*, additional ring-like structures (H-ring and T-ring) have also been observed in the periplasmic space (7). The rotary motor is powered by the transmembrane ion flux (8–10), and the interaction between the C-ring and the stators is thought to be essential in torque generation (11, 12). The probability of motor rotating clockwise (CW), the CW bias, is adjusted by the chemotaxis signaling pathway (13). In E. coli and *Salmonella*, the response regulator CheY, when phosphorylated (yielding CheY-P), could bind to the C-ring and promote CW rotation of the motor (14, 15). However, in B. subtilis, CheY-P binding to the motor promotes counterclockwise (CCW) rotation (16). The chemotaxis signaling pathways present inverted characteristics in B. subtilis and E. coli; that is, attractants would induce increased and decreased levels of CheY-P in B. subtilis and E. coli, respectively (13, 16).

Structure biology techniques with higher resolution than optical microscopy, such as cryo-electron microscopy (cryo-EM) and cryo-electron tomography (cryo-ET), have been widely used to investigate the structural features of flagellar motors (5). In early cryo-EM structural analysis, the symmetry mismatch between the MS-ring (~26-fold) and the C-ring (~34-fold) of the *Salmonella* flagellar motor brought conundrums for arrangement, torque generation, and other characteristics of the motor (17). Furthermore, the turnover and adaptation of C-ring subunits increased the complexity of arrangement of the motor structure (18–22). With recent advances in cryo-EM techniques, the puzzle of symmetry mismatch between the MS-ring and the C-ring seemed resolved. Although the symmetry mismatch was still present (23, 24), it was much smaller than that previously reported (25–27). For E. coli and *Salmonella*, the native MS-ring is composed of 34 FliF subunits with no symmetry variation, whereas the symmetry of the C-ring varies from 32- to 36-fold with a peak at 34-fold (24). *In situ* visualization of the C-ring in motors also showed a 34-fold symmetry by cryo-ET, although measured in Vibrio alginolyticus (28). Furthermore, flagellar motors composed of FliF-FliG fusion proteins can assemble and rotate with normal-looking basal bodies (29–32), indicating that FliG and FliF probably exist in a 1:1 stoichiometry (33). Another piece of evidence for FliG stoichiometry is the stability of FliF and FliG subunits after full assembly of a functional flagellar motor. Unlike FliM and FliN subunits with adaptive turnover (18–22), the FliF and FliG molecules assembled to the rotating motor were stable without recovery of fluorescence in fluorescence recovery after photobleaching (FRAP) experiments (19, 34). Therefore, the small variation in C-ring symmetry indicates limited flexibility in FliG ring assembly around the MS ring, and the stoichiometry of FliG in a functional flagellar motor could be estimated with a mean value of 34.

The E. coli C-ring has been shown to undergo adaptive remodeling in response to changes in motor directional bias. Nevertheless, it remains unclear how the C-ring accomplishes this adaptive remodeling. Recent cryo-EM structural analysis indicated that both CCW- and CW-locked motors showed highly similar symmetry distributions of the C ring, with a peak at ~34-fold (24, 32), which was in agreement with the previous observation of electron cryomicrographs (26, 27). Note that the C-ring diameters of the CCW and CW motors are nearly the same in cryo-EM images (32). However, adaptive remodeling of the motor results in significantly larger copy numbers of FliM and FliN subunits in the CCW state than in the CW state (20–22), whereas the spacing between adjacent FliM subunits seems similar in CCW and CW motors by fluorescence anisotropy analysis (35). If FliM monomers form an intact ring, the ratio of CCW/CW ring diameter should be ~1.3 according to the copy number difference (20). This raises the questions of why such changes in stoichiometry and ring size have never been observed by cryo-EM and how the C-ring would accommodate a fixed number of FliG molecules with alterable numbers of FliM and FliN molecules. Here, we investigated the stoichiometry and chemotactic function of FliM in CCW- and CW-locked motors of E. coli by fluorescence labeling, obtaining an unexpected arrangement of the C-ring subunit.

## RESULTS

### Robustness of the FliG copy number in functional motors.

**FIG 1:**
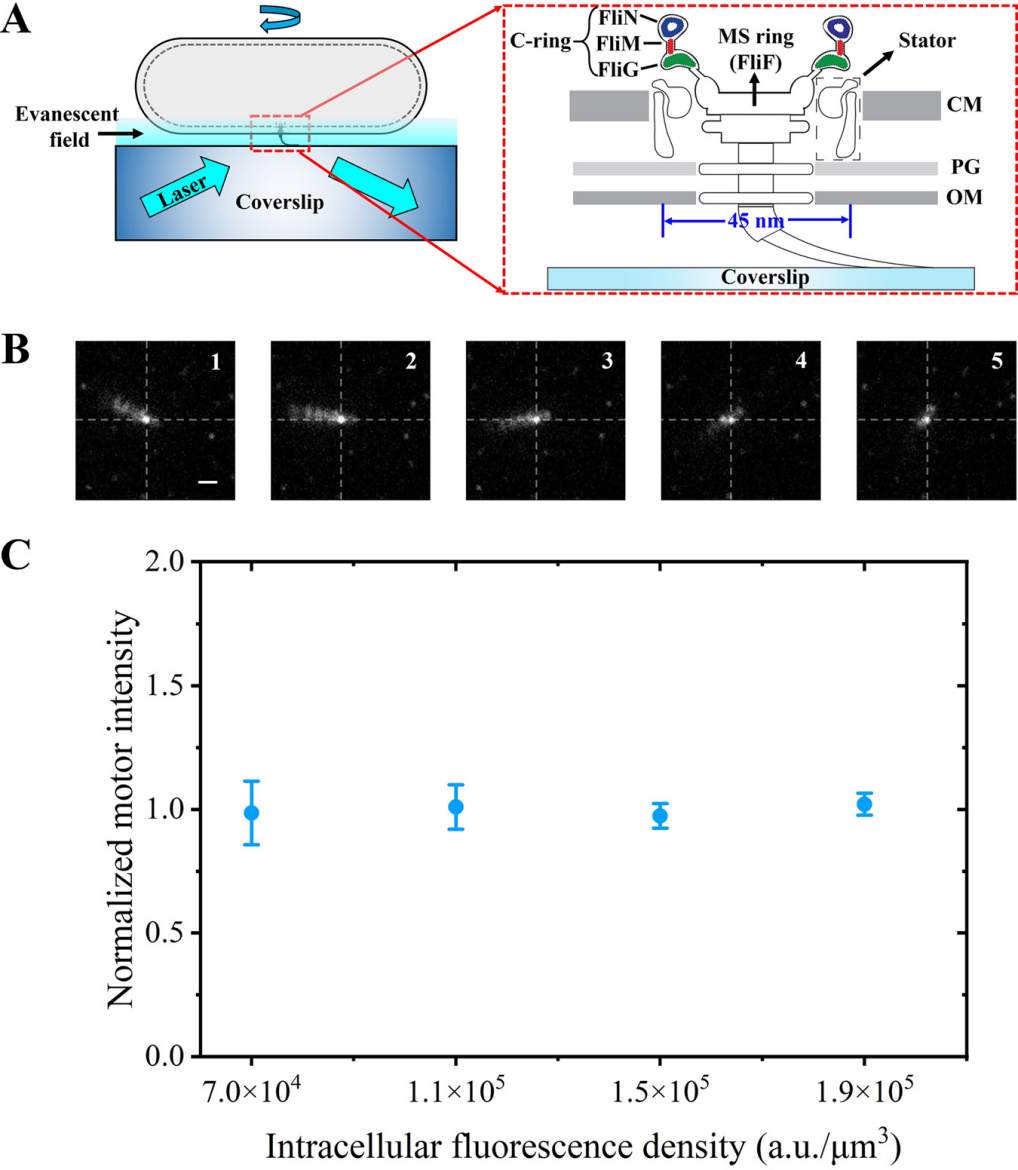
Tethered-cell experiments and robustness of FliG copy number in the motor. (A) Tethered-cell assay under TIRF illumination. (Inset) Schematic of the C-ring in the rotary motor (CM, cytoplasmic membrane; PG, peptidoglycan layer; OM, outer membrane). (B) Initial 5 frames of TIRF fluorescence images of a rotating motor containing mEGFP-FliG (bar, 1 μm). The intersection of two dashed lines indicates the center of rotation (i.e., a functional motor). (C) Robustness of the motor fluorescence intensity (normalized by the mean value) at various intracellular fluorescence densities (20 individual cells) corresponding to various expression levels of mEGFP-FliG. Error bars represent the SEM.

The strains and plasmids used in this study are listed in Table 1 (see Materials and Methods for details). By using the isopropyl-β-d-thiogalactopyranoside (mEGFP)-inducible plasmid pTrc99a, monomeric enhanced green fluorescent protein (mEGFP) (i.e., EGFP^A206K^) was genetically fused to the N terminus of FliG, yielding mEGFP-FliG. The strain AT1 (Δ*fliC*, *fliG*) carrying two plasmids pAT3 (*pTrc99a-megfp-fliG*) and pKAF131 (*pACYC184-fliC*^sticky^) was used for tethered-cell experiments (Fig. 1A). For the maturation of mEGFP molecules, motile cells were preincubated at 37°C for 45 min in motility buffer to minimize the additional expression of mEGFP molecules and restrict cell growth (36). Therefore, the concentration of mEGFP molecules should be roughly constant during incubation. The motor fluorescence was recorded under total internal reflection fluorescence (TIRF) illumination for 2 s, after which the intracellular fluorescence of the same cell was captured under epifluorescence illumination. The fluorescence intensity of individual motors for each frame was calculated, and the background fluorescence intensity was subtracted (see Materials and Methods for details). The final motor intensity was calculated as the mean value for the initial 5 frames (Fig. 1B). The intracellular fluorescence density, defined as the ratio of the net intracellular fluorescence to the volume of the cell, has been confirmed to be approximatively proportional to the intracellular concentration of fluorescent proteins in individual cells (37). The motor fluorescence intensity was nearly constant regardless of the changes in the intracellular fluorescence density (Fig. 1C), confirming the robustness of the FliG copy number in functional motors at various FliG expression levels. Therefore, the FliG copy number was restricted by the intrinsic structure of the motor, namely, the sequential assembly of the C-ring around the MS-ring (containing 34 FliF) rather than the intracellular concentration of FliG molecules. Our measurements were consistent with the previous finding that the stoichiometry of FliG in a functional flagellar motor has a mean value of 34, the same as the copy number of FliF.

**TABLE 1:** List of cell strains and plasmids used in the study

Strain or Plasmid	Description	Source or Reference
Strain		
RP437	Wild-type	71
GL2	Δ*fliC*, *fliM*, *cheY*	53
AT1	Δ*fliC*, *fliG*	This work
XL2	Δ*fliC*, *cheY*, *cheB*, *cheZ*	37
GL1	Δ*fliC*, *fliM*	53
HCB1357 (VS116)	Δ*flhC*	34
Plasmid		
pAT1	*pBAD33-cheY-megfp*	37
pAT2	*pTrc99a-fliM-megfp*	This work
pAT3	*pTrc99a-megfp-fliG*	This work
pAT4	*pBAD33-fliC*(ST)-*cheY13DK106YW*	This work
pAT5	*pBAD33-megfp*	This work
pKAF131	*pACYC184-fliC*(ST)^*a*^	38
pFD313	*pBR322 based, fliC*(ST)	72

a ST, sticky phenotype.

### FliM copy numbers in CCW- and CW-locked motors.

**FIG 2:**
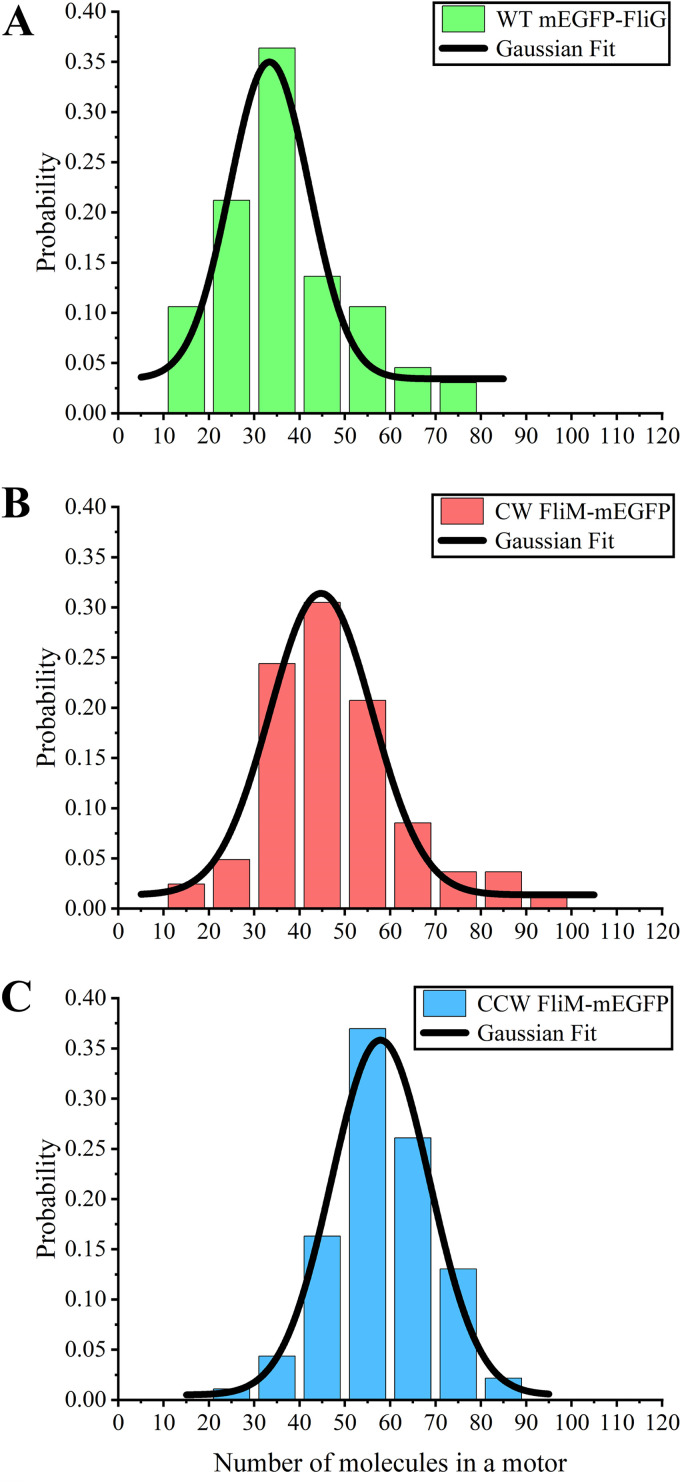
Distribution of the copy number of mEGFP-FliG in (A) wild-type (WT) motors (66 cells) and FliM-mEGFP in (B) CW- (82 cells) and (C) CCW-locked motors (92 cells).

We aimed to measure the FliM copy numbers in individual motors rotating exclusively CCW or CW by utilizing the fluorescence intensity of the motor composed of mEGFP-FliG as a reference. Monomeric EGFP was genetically fused to the C terminus of FliM, yielding FliM-mEGFP. The strain GL2 (Δ*fliC*, *fliM*, *cheY*) carrying pAT2 (*pTrc99a-fliM-megfp*) and pKAF131 was used for observing CCW-locked motors of tethered cells. For CW-locked motors, we constructed the plasmid pAT4 to simultaneously express the sticky phenotype of FliC and the mutant CheY^13DK106YW^ that was constitutively active even without phosphorylation (38). GL2 was transformed with pAT2 and pAT4, and excess arabinose was added to induce the overexpression of the CheY^13DK106YW^ mutant for CW-locked motors. First, by performing tethered-cell experiments, the photobleaching profile of individual motors containing mEGFP-FliG molecules was fitted to a single exponential function (39) as follows:

(1) $$I(t)\;=\;I_{\text{0}}\;\times \;e^{–\mathrm{kt}}$$

where *I*(*t*) is the fluorescence intensity of a motor at laser exposure time *t*, *k* is the photobleaching rate constant, and *I*_0_ is the initial intensity of the motor. The distribution of initial intensities (*I*_0_) for the mEGFP-FliG motors was fitted to a Gaussian curve to obtain the average intensity (*I*_G_), which corresponded to the overall brightness emitted from all of the mEGFP-FliG molecules in the C-ring. Subsequently, under identical illumination and imaging conditions, the fluorescence intensities of CCW- and CW-locked motors composed of FliM-mEGFP molecules were recorded. For each photobleaching profile, the number of FliM molecules (*N*_FliM_) was estimated by

(2) $$N_{\text{FliM}}\;=\;\frac{I_{\text{0}}}{I_{\text{G}}}\;\times \;\text{34}$$

where *I*_0_ is the fitted initial intensity, and 34 is the estimated average number of FliG subunits in the C-ring. Surprisingly, the copy numbers of FliM-mEGFP in CW- and CCW-locked motors exhibited a Gaussian distribution peaking at 45 ± 11 and 58 ± 11 (errors denote standard deviation [SD]), respectively (Fig. 2), which were both larger than the copy number of FliG molecules in the C-ring. The ratio of the FliM copy numbers in CCW- versus CW-locked motors was ~1.29, which was in good agreement with the previous measurement (20). We noted that the previously measured FliM copy numbers in the CCW- and CW-locked motors were ~44 and 34, respectively, on the assumption that FliM in CW-locked motors had a copy number of 34 (20). CW-locked motors contain fewer FliM molecules than CCW-locked motors because of the adaptive remodeling of the motor (21). We further confirmed that the brightness emitted from a mEGFP cluster was comparable whether fused to the N terminus or the C terminus of the target protein by comparing the mean intracellular fluorescence density for mEGFP-FliG induced by 130 μM IPTG and FliM-mEGFP induced by 100 μM IPTG, obtaining a ratio in agreement with the value from Western blot analysis (further details in Text S1 in the supplemental material). In addition, using the strain GL1 (Δ*fliC*, *fliM*) transformed with pAT2 and pKAF131, we performed tethered-cell experiments to eliminate the possible dependence of the quantum efficiency of FliM-mEGFP molecules on the motor switch state (see Fig. S3 in the supplemental material).

10.1128/mbio.00189-23.1 Text S1 Comparison of the overall fluorescence efficiency of mEGFP fusions. Detection of photons emitted from individual mEGFP molecules. Download Text S1, DOCX file, 0.02 MB. Copyright © 2023 Tao et al. 2023 Tao et al. https://creativecommons.org/licenses/by/4.0/ This content is distributed under the terms of the Creative Commons Attribution 4.0 International license.

10.1128/mbio.00189-23.4 FIG S3 Distribution of the ratio of the motor brightness in the CCW frame to that in the adjacent CW frame when the motor switches direction. The photobleaching profile of a motor was fitted to a single exponential function (I(t) = I0 × e−kt), and the brightness in each frame was divided by exp(−*kt*) to compensate for fluorescence bleaching. The Gaussian fit to the distribution peaked at 1.02 ± 0.06. Download FIG S3, TIF file, 6.7 MB. Copyright © 2023 Tao et al. 2023 Tao et al. https://creativecommons.org/licenses/by/4.0/ This content is distributed under the terms of the Creative Commons Attribution 4.0 International license.

### Numbers of CheY-P molecules binding to a CW spinning motor.

**FIG 3:**
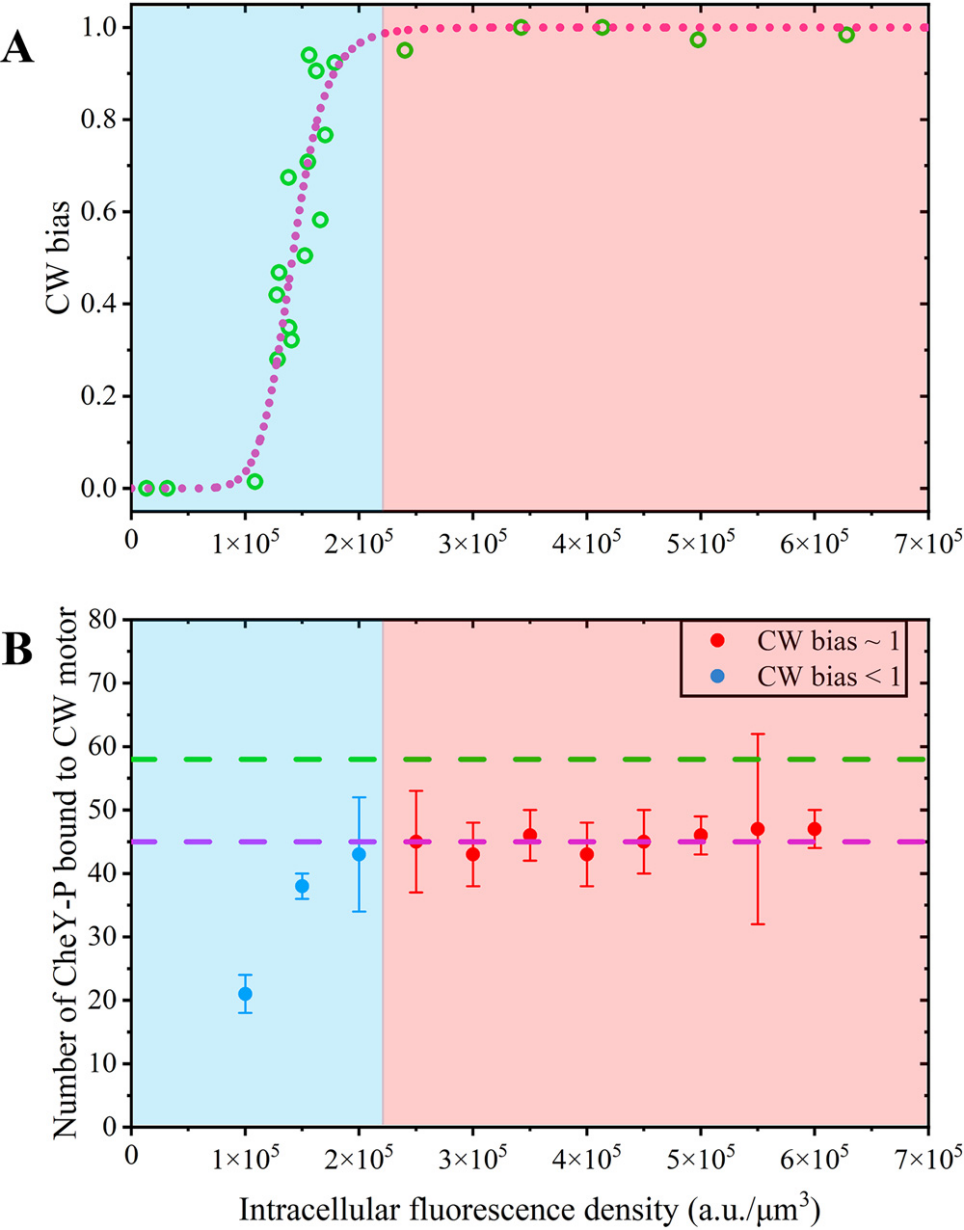
Number of CheY-P molecules bound to a CW spinning motor. The blue shaded area corresponds to the circumstance that CW bias is <1, and the red shaded area corresponds to the circumstance that CW bias is ~1. (A) The CW bias versus the intracellular fluorescence density of individual cells containing CheY-P-mEGFP. The green circles are experimental data, and the magenta dotted line shows the fit with a Hill function. (B) The number of CheY-P molecules bound to a CW rotating motor versus the intracellular fluorescence density. Error bars represent the SEM. The green and violet dashed lines denote the copy numbers of FliM in CCW- and CW-locked motors, respectively.

**FIG 4:**
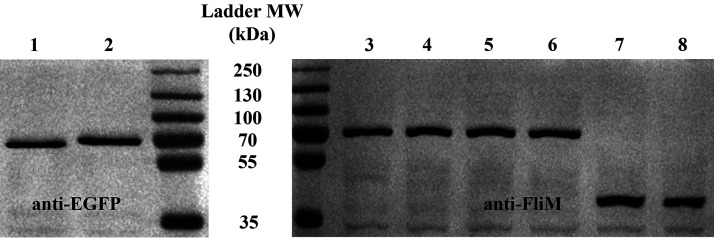
Representative chemiluminescence images with different primary antibodies (left, anti-EGFP; right, anti-FliM). Molecular weight values (kDa) are labeled on the middle column of the panel. Lane 1, mEGFP-FliG induced by 130 μM IPTG. Lanes 2 to 6, FliM-mEGFP induced by 100 μM IPTG. Lanes 7 and 8, FliM produced in the wild-type strain.

FliM molecules of a functional motor play a crucial role in the chemotaxis signaling pathway because binding of the response regulator CheY-P to FliM promotes CW rotation of the motor (14, 40, 41). Our measurements suggest that there are more FliM molecules in functional motors than previous estimation, raising the question of whether all of the FliM molecules could be bound by CheY-P molecules. Here, we used the strain XL2 (Δ*fliC*, *cheY*, *cheB*, *cheZ*) carrying pAT1 (*pBAD33-cheY-megfp*) and pFD313 (*pBR322-fliC*^sticky^) to observe the binding of CheY-P-mEGFP to a rotating motor under identical illumination and imaging conditions with measurements of the motor fluorescence intensity of mEGFP-FliG and FliM-mEGFP. This strain promotes the phosphorylation of CheY and inhibits the dephosphorylation of CheY-P; hence, all of the intracellular CheY-mEGFP molecules are essentially phosphorylated with an analogous physiological function to wild-type (WT) CheY-P (42, 43). The motor CW bias was analyzed with a 1-min time sequence of bright-field images. Then, the motor fluorescence of individual tethered cells was recorded under TIRF illumination for 5 s, after which the intracellular fluorescence of the same cell was captured under epifluorescence illumination. The profile of the CW bias versus intracellular fluorescence density fell onto a sigmoid curve with a Hill coefficient of ~9.6 (Fig. 3A), demonstrating a similar ultrasensitivity to a previous measurement using a bead assay (42). The off-rate of CheY-P bound to FliM was estimated to be >15 s^−1^ (44), which was more than 750 times larger than the off-rate (~0.02 s^−1^) of FliM bound to the C-ring (20). Quickly exchanging components, such as CheY-P bound to the C-ring, are not suitable for the photobleaching fit method (18, 22, 39). Furthermore, the duration of a motor staying in the CW state is sometimes shorter than the photobleaching time of motor intensity, especially for motors with CW bias close to 0. Therefore, instead of the photobleaching fit using the CW frames, TIRF movies were manually analyzed by eye to find the initial CW interval longer than 5 frames, and the fluorescence intensity of CheY-mEGFP bound to the motor was calculated as the mean value for the 2nd to 4th frames of the CW interval. We performed a direct comparison between the intensities of motors containing mEGFP-FliG found by the two methods as follows: one is the photobleaching fit, and the other is the mean of the initial frames. As shown in Fig. S4 in the supplemental material, both methods lead to equivalent results. Moreover, we also detected the number of photons emitted from the three types of mEGFP fusions used in the present study at the single-molecule level. We found that individual mEGFP molecules emitted the same number of photons regardless of the type of fusion (see Text S1 and Fig. S5 in the supplemental material). This provided additional support for reliable counting by using different mEGFP fusions. Using the same reference as *N*_FliM_ (equation 2), the motor fluorescence intensity was used to estimate the number of CheY-P molecules bound to the motor, which was then plotted versus the corresponding intracellular fluorescence density (Fig. 4B). Note that the intracellular fluorescence should be corrected by the fraction of bleached mEGFP during the TIRF illumination; however, applying this correction factor only results in a scale-up of horizontal coordinates that are originally in arbitrary units, thus neglecting that the correction should not affect the presented information of the profile. With the increase in CW bias from 0 to 1 as the intracellular fluorescence density increases, the number of CheY-P molecules bound to a CW spinning motor increases rapidly and nearly saturates when the intracellular fluorescence density falls into the region where the CW bias is 1. The FliM occupancy with CheY-P varies from 0.36 (21/58; CW bias, ~0) to 1 (45/45; CW bias, ~1) in CW rotating motors, indicating that not all of the FliM subunits are required to be bound by CheY-P molecules to induce CW rotation. This was consistent with the Monod-Wyman-Changeux (MWC) model (45, 46) or the conformational spread model (47–49) of the flagellar switch. Therefore, all of the FliM molecules in the C-ring can bind CheY-P. Looking from a different perspective, the saturated number of CheY-P molecules bound to a CW rotating motor that we measured here provided an additional piece of evidence that the copy number of FliM in CW-locked motors is 45.

10.1128/mbio.00189-23.5 FIG S4 Comparison between the initial motor intensities found by two different methods. Motors containing mEGFP-FliG were analyzed for comparison. (A) Distributions of the initial motor intensity. The upper part shows the results analyzed by exponential fitting of the photobleaching profile, while the lower part shows the results analyzed as the mean value for the initial three frames of steady fluorescence emission. The Gaussian fits for the upper and lower parts peaked at 2,661 ± 894 and 2,641 ± 1,064 (mean ± SD), respectively. (B) Distribution of the relative result bias. For each individual motor, the relative result bias was defined as (*A* − *E*)/*E*, where *A* is the result analyzed as the mean value of the initial three frames and *E* is the result analyzed by exponential fitting of the photobleaching profile. The Gaussian fit to the distribution peaked at −0.001 ± 0.087 (mean ± SD). Download FIG S4, TIF file, 0.9 MB. Copyright © 2023 Tao et al. 2023 Tao et al. https://creativecommons.org/licenses/by/4.0/ This content is distributed under the terms of the Creative Commons Attribution 4.0 International license.

10.1128/mbio.00189-23.6 FIG S5 Distributions of photon counts emitted from individual fused mEGFP molecules per frame. For the three types of fused mEGFP molecules, the first peak (indicated by the red arrow) of the distributions exhibited similar values of photons. The peak positions are 166 ± 30, 165 ± 32, and 163 ± 27 (mean ± SD) photons for mEGFP-FliG, FliM-mEGFP, and CheY-mEGFP, respectively. PDF, probability density function. Download FIG S5, TIF file, 0.4 MB. Copyright © 2023 Tao et al. 2023 Tao et al. https://creativecommons.org/licenses/by/4.0/ This content is distributed under the terms of the Creative Commons Attribution 4.0 International license.

### Function and expression levels of mEGFP-fused motor proteins.

Flagellar motors composed of FliM molecules with C-terminal fusions of homologous fluorescent proteins have been confirmed to retain CW biases, switching rates, and rotating speeds similar to those of the wild-type motor (21, 43). The functions of FliG molecules are partially affected by N-terminal fused GFP (50). In brief, GFP-FliG molecules are stable with negligible degradation and are functional, with motors exhibiting lower CW biases and switching frequencies than those of wild-type motors (50). We observed similar characteristics for mEGFP-fused molecules in this study. Therefore, both FliM-mEGFP and mEGFP-FliG are able to compose intact flagellar motors that are functional for further stoichiometry analysis.

For stoichiometry, the expression of FliM-mEGFP and mEGFP-FliG was induced to the wild-type levels according to quantitative Western blot analysis (Fig. 4). By using anti-FliM antibody, the amount of FliM-mEGFP induced by 100 μM IPTG was compared with the amount of FliM produced in the wild-type strain, obtaining a ratio (FliM-mEGFP/FliM) of 0.97 ± 0.36 (mean ± SD from 12 individual bands). Furthermore, the intracellular levels of FliG and FliM proteins in the wild-type strain were measured in a previous study, obtaining a ratio (FliG/FliM) of ~0.71 (51). In this work, the amount of mEGFP-FliG induced by 130 μM IPTG and the amount of FliM-mEGFP induced by 100 μM IPTG were compared by using anti-EGFP antibody, obtaining a ratio of 0.74 ± 0.37 (mean ± SD from 4 individual bands), which was in good agreement with the previous measurement (51).

## DISCUSSION

The conundrum of symmetry mismatch between the MS-ring and the C-ring in E. coli and *Salmonella* motors seems resolved by recent cryo-EM analysis (23, 24); that is, both the MS-ring and the C-ring have 34-fold symmetry. Furthermore, flagellar motors composed of FliF-FliG fusion proteins can assemble and rotate with normal-looking basal bodies (29–32), and FliF requires FliG to facilitate MS-ring formation in the cytoplasmic membrane (52). FliF and FliG molecules in the motor are stabilized without protein exchange once a functional motor is fully assembled (19, 34). In the present study, we demonstrated the robustness of the FliG copy number of the motor at various intracellular concentrations of FliG, suggesting that the assembly of FliG subunits was restricted by the sequential assembly of the C-ring around the MS-ring composed of 34 FliF molecules. This further confirmed that the mean value of the FliG copy number is ~34 in the C-ring of the E. coli flagellar motor. In contrast, previous measurements of motor fluorescence (with FliM-eGFP) as a function of cytoplasmic fluorescence showed that the number of FliM molecules in a motor increased as the cytoplasmic FliM concentration increased (53). Thereafter, we directly compared the fluorescence intensity of motors containing mEGFP-FliG and FliM-mEGFP under identical experimental conditions. By using an FliG stoichiometry of 34 as a reference, the distributions of FliM copy numbers peaked at 58 and 45 for the CCW- and CW-locked motors, respectively. Therefore, the ratio of FliM to FliG numbers in CW-locked motor was ~1.32, and the ratio of FliM numbers in CCW-locked to CW-locked motors was ~1.29. A previous study showed a similar ratio of FliM numbers in CCW-locked to CW-locked motors of ~1.3 and proposed that the FliM copy numbers in the CCW- and CW-locked motors were ~44 and 34, respectively, based on the assumption that FliM in CW-locked motors had a copy number of 34 (20) because the symmetry of the C-ring in the CW-locked *Salmonella* motor peaked at 34-fold in previous cryo-EM studies (27). Note that the symmetry of the C-ring, which was analyzed by looking at end-on view images of the ring, always peaked at 34-fold regardless of the rotational direction in recent cryo-EM analysis (24, 32), suggesting that this symmetry probably corresponded to the copy number of FliG subunits in the C-ring or that it corresponded to the shrunken FliM ring (see below).

In early protein structural analysis, the middle domain of FliG (FliG_M_) and the C-terminal domain of FliG (FliG_C_) were both supposed to interact with FliM, suggesting that there might be two FliM binding sites per FliG (54–56). However, the interaction between FliG_M_ and neighboring FliG_C_, which played a crucial role in the organization and ring formation of FliG subunits in the motor, was proven in later reports (31, 57, 58). Therefore, the direct binding of FliM to FliG_C_, which would interrupt the array of FliG_C_-FliG_M_ interactions, seemed unlikely (59). Alternatively, recent crystal structural analyses suggested that the middle domain of FliM (FliM_M_) interacts with FliG_M_ to form a complex with a stoichiometry of 1 FliG to 1 FliM in the basal body of the motor (32, 59–61). This raises the question of how the C-ring would accommodate extra FliM subunits if the number of FliM binding sites is equal to the number of FliG molecules. Assuming each FliM needs to be bound to an FliG, the maximum copy number of FliM should be 34, probably corresponding to the CCW state. In this case, the copy number of FliM could be estimated to be ~26 in the CW state, which would result in 8 gaps in the FliM-FliN track. However, the possible gaps have not been observed by cryo-EM (24, 27, 32). Our measurements here showed that the C-ring contains more FliM subunits than FliG subunits for both CCW and CW states. A recent report proposed a “bridging” model in which each FliG_M_ domain rests on a FliM_M_ domain, and each FliG_C_ domain stacks onto the FliG_M_ domain of an adjacent subunit, while the segment linking FliG_M_ to FliG_C_ could be in either an extended or a helical conformation (31). The extended conformation would allow adjacent FliG subunits to bridge over an extra FliM subunit, which mainly interacts with the adjacent FliM subunits and does not interact strongly with FliG. The maximum copy number of FliM subunits could be estimated to be 68 if all of the linking segments exist in the extended conformation. Alternatively, other models envisioning a fixed-diameter C-ring could tolerate binding of “extra” FliM subunits that are not incorporated into the ring (62).

It was still unclear why the differences in stoichiometry and size of the C-ring between the CCW and CW states were not observed by cryo-EM. The symmetry and diameter of the C-ring are similar for the CCW and CW states in cryo-EM studies (24, 32), suggesting that there are possible changes in protein conformation rather than composition when the motor switches direction. Note that the MotA/MotB stator unit exchange took place in E. coli motors (62), and the stator complexes associated with the basal body seemed lost during sample purification for cryo-EM (59). A recent review proposed the possibility that during the purification of the basal body for cryo-EM, FliM/FliN subunits might leave the basal body without replacement by cytoplasmic FliM/FliN molecules (62). If so, cryo-EM images would reflect the native stable structure after ring shrinkage. Assuming that FliM molecules interacting strongly with FliG molecules are much more stable than those “extra” FliM molecules either below the extended “bridge” or not incorporated into the ring, the latter would leave the C-ring during purification, and FliG and FliM would exist in a 1:1 stoichiometry in the shrunken ring. In the present study, we found that both the CCW and CW FliM numbers were larger than the FliG number. This provided a possibility for ring shrinkage.

In previous studies, another component of the C-ring, FliN, has also been confirmed to undergo turnover and adaptive remodeling (19, 22). The ratio of the mean FliN copy numbers in CCW-locked versus CW-locked motors is similar to the value found for FliM (20, 22), suggesting that FliM and FliN might form a coexchange unit. Previous studies have proposed two models of the FliM-FliN complex. The first model with stoichiometry of FliM_1_:FliN_4_ proposed that four FliN monomers form a tetramer at the base of the C-ring (15, 63, 64). The second model with FliM_1_:FliN_3_ predicted that a FliM:FliN heterodimer and a FliN homodimer form a spiral base of the C-ring (65). Recent cryo-ET structural analyses agreed well with the FliM_1_:FliN_3_ model because of the absence of additional density for a fourth FliN molecule (28, 66). Our measurements of FliM copy numbers in the present study and the previously measured copy numbers of FliN in CCW- and CW-locked motors (22) suggest a composition of complex closer to FliM_1_:FliN_3_ than FliM_1_:FliN_4_. Nevertheless, *in situ* structural analysis of the C-ring with higher resolution would be necessary to verify the model in future studies.

Here, we demonstrated that all of the FliM molecules in the C-ring could bind CheY-P. Note that the interaction between CheY-P and FliN also plays a critical role in motor switching (15). A recent report suggested that CheY interacts with FliM_N_, FliN, and FliM_M_ during motor switching (41). In a proposed model, CheY would first be bound to a flexible high-affinity site of FliM (FliM_N_), subsequently to FliN, and then to a separate, low-affinity active site on FliM (FliM_M_) that would promote CW rotation of the motor (41). In the conformation spread model of the flagellar switch (48), the C-ring is modeled as a ring of a fixed number of subunits, each of which has a binding site for a CheY-P molecule with affinity depending on its conformational state. CheY-P binding changes the free energy level of the subunits. There is a coupling energy between adjacent subunits that favors the same conformations for adjacent subunits. The conformation spread model can well describe the ultrasensitivity of the flagellar switch but is not able to describe motor adaptive remodeling by simply varying the number of subunits. Our measurements here provided new insights to update the conformation spread model. The subunits in the model with a fixed number correspond to individual FliG molecules, and FliM molecules that interact strongly with FliG would bind CheY-P to modulate the energy level of the subunits. FliM_N_ likely acts as a flexible “fishing line” that serves to bind and concentrate CheY-P (41). Therefore, more FliM molecules recruit larger numbers of CheY-P around the C-ring. Due to the flexibility of FliM_N_, CheY-P bound to FliM_N_ of the extra FliM molecules might be able to interact with neighboring FliN/FliM_M_ that interact strongly with FliG, thus increasing motor sensitivity to CheY-P concentration. This is consistent with previous experimental findings (21).

Cryo-ET was utilized to visualize *in situ* CCW and CW motor C-ring structures of Vibrio alginolyticus (28) and Borrelia burgdorferi (66). In V. alginolyticus and B. burgdorferi, the C-ring maintained 34-fold and 46-fold symmetries (in both CCW and CW states), respectively, suggesting that the C-ring protein composition probably remained constant for the two species, although there were some extra densities associated with the C-ring (28, 66). These findings were different from previous fluorescence studies in which the FliM/FliN subunits underwent copy number changes in E. coli and *Salmonella* motors (20–22). However, there have been no reports of *in situ* cryo-ET studies of CCW and CW motor C-ring structures in E. coli or *Salmonella* to date. Therefore, it remains unclear whether C-ring protein exchange takes place in bacterial species other than E. coli or *Salmonella*, especially for those in which the stoichiometry of the C-ring protein composition remained constant in cryo-ET studies. It was interesting to note that E. coli and *Salmonella* stator complexes were not observed by *in situ* cryo-ET (3, 67), probably because stator complexes in these two species were dynamic and the low viscous loads in sample preparation led to stator dissociation (62). However, some other species with large periplasmic structures, such as *Vibrio*, have shown well-defined and clearly imaged rings of stator units by *in situ* cryo-ET (28, 67). This raises the possibility that the characteristics of stator unit exchange are different or that the stator units do not exchange in these species. A possible speculation is that motors with a stable composition should be crucial for generating continuous and sufficient torque, especially for bacterial species possessing a single polar flagellum such as *Vibrio*. In contrast, for E. coli and *Salmonella*, the run-and-tumble behavior depends on the “cooperation” of several flagella around the cell body (2). Stator unit exchange allows for rapid motor adaptation to changes in energy availability and load conditions, whereas FliM/FliN unit exchange would adjust the chemotactic sensitivity by adaptive remodeling of the C-ring. These different characteristics between bacterial species might correspond to different survival and evolutionary strategies.

## MATERIALS AND METHODS

### Strains and plasmids.

The E. coli strains and plasmids used in this study are listed in Table 1. All strains are derivatives of the E. coli K12 strain RP437. The plasmids pAT1, pAT4, and pAT5 are based on the vector pBAD33 with an arabinose-inducible promoter (68), and pAT2 and pAT3 are based on the vector pTrc99a with an isopropyl-β-d-thiogalactopyranoside (IPTG)-inducible promoter. For the observation of FliM-mEGFP in CCW-locked motors, GL2 (Δ*fliC*, *fliM*, *cheY*) transformed with pAT2 and pKAF131 was used. For the observation of FliM-mEGFP in CW-locked motors, GL2 transformed with pAT2 and pAT4 was used. For the observation of mEGFP-FliG in the wild-type motors, AT1 (Δ*fliC*, *fliG*) transformed with pAT3 and pKAF131 was used. For the observation and comparison of FliM-mEGFP brightness in switching motors, GL1 (Δ*fliC*, *fliM*) transformed with pAT2 and pKAF131 was used. For the observation of CheY-mEGFP in CW spinning motors, XL2 (Δ*fliC*, *cheY*, *cheB*, *cheZ*) transformed with pAT1 and pFD313 was used. To estimate the background correction factor for motor intensity analysis, XL2 transformed with pAT5 and pFD313 was used. For the detection of photons emitted from single CheY-mEGFP molecules, HCB1357 (Δ*flhC*) transformed with pAT1 was used. For the detection of photons emitted from single FliM-mEGFP molecules, HCB1357 transformed with pAT2 was used. For the detection of photons emitted from single mEGFP-FliG molecules, HCB1357 transformed with pAT3 was used. The wild-type strain RP437 was used in Western blotting experiments as the control group to determine the expression level of FliM.

### Cell culture and preparation.

All strains were grown in tryptone broth (1% tryptone, 0.5% NaCl) at 33°C to mid-log phase (optical density at 600 nm [OD_600_] = 0.6). When needed, ampicillin and chloramphenicol were added to final concentrations of 100 and 25 μg/mL, respectively. For stoichiometry measurements, we grew the FliM-mEGFP and mEGFP-FliG strains with 100 and 130 μM IPTG to induce wild-type levels of protein expression, respectively. For CW-locked motors, an additional 0.1% arabinose was added to induce the expression of sticky FliC and excessive CheY^13DK106YW^. For the strain XL2 carrying pAT1 and pFD313, 0.001 to 0.005% arabinose was added to cover the whole range of motor switching behaviors. For the strain XL2 carrying pAT5 and pFD313, 0.01% arabinose was added to induce mEGFP expression. For fluorescence observation, cells were washed twice with motility buffer (0.01 M potassium phosphate, 10^−4^ M EDTA, 0.067 M NaCl, pH 7.0), resuspended in lightproof tubes, and incubated in motility buffer at 37°C for 45 min. More than 90% of mEGFP molecules matured after incubation (36). To shorten flagellar filaments, resuspended cells were forced to pass through narrow polyethylene tubing (0.58-mm inner diameter) connected between two syringes more than 100 times. The sample chamber was precleaned and constructed as described previously (37). For tethered-cell experiments, cells with sheared filaments were harvested by centrifugation, resuspended in motility buffer, and loaded into the chamber for 15 min. Then, 400 μL motility buffer was gently injected through the chamber to remove unattached cells. The cell culture and sample preparation were kept in dark environments to avoid fluorescence excitation before data acquisition.

### Microscopy and image acquisition.

We used a Nikon Eclipse Ti2 TIRF microscope to observe the fluorescence emitted from mEGFP molecules. For the excitation of fluorescence, a fiber-pigtailed laser beam (Sapphire 488 FP; Coherent) was reflected by a primary dichroic mirror (Chroma) and focused on the back focal plane of the TIRF objective lens (100× Apo TIRF, NA 1.49; Nikon). To measure the motor fluorescence of tethered cells, the angle of incidence was adjusted to generate an evanescent laser field with a characteristic decay length of 100 nm. A halogen lamp was used for bright-field illumination with a longpass glass filter (Thorlabs) to minimize the potential excitation of fluorescent molecules. The fluorescence emitted from the whole cell was captured under epifluorescence illumination. All of the fluorescence images of tethered cells were recorded at 65 nm per pixel with a back-illuminated scientific complementary metal oxide semiconductor (sCMOS) camera (Dhyana 400BSI; Tucsen Photonics) at 20 frames/s. The bright-field images for analyzing the CW bias were recorded at 64 frames/s.

### Intracellular fluorescence density.

Fluorescence emitted from the whole cell was recorded under epifluorescence illumination. The net fluorescence was confirmed to be proportional to the quantity of mature fluorescent proteins in a previous study (36). Therefore, we defined the intracellular fluorescence density as the ratio of the net fluorescence to the cell volume, which has been confirmed to be approximatively proportional to the intracellular concentration of fluorescent proteins (37). The length of the cell was determined with the bright-field image, and the shape of an E. coli cell was approximated as a cylinder with a diameter of 0.8 μm with two hemispherical end caps to estimate the cell volume (37, 69). The net fluorescence was calculated to capture most of the out-of-focus light (36) (see Fig. S2 in the supplemental material).

10.1128/mbio.00189-23.3 FIG S2 Quantification of the net fluorescence emitted from the whole cell. Two windows were used for quantification. The length of the rectangular window (red dots) was set as the cell length plus 1.6 μm, and the width was set as 4 μm to capture most of the out-of-focus light. The side length of the square window (yellow lines) was set as the cell length plus 2.6 μm. The mean background intensity per pixel was calculated as the mean intensity value of pixels inside the square window while outside the rectangular window. The net fluorescence was quantified as the sum of all pixel intensity values within the rectangular window after subtraction of the mean background intensity from each pixel value. Download FIG S2, EPS file, 3.7 MB. Copyright © 2023 Tao et al. 2023 Tao et al. https://creativecommons.org/licenses/by/4.0/ This content is distributed under the terms of the Creative Commons Attribution 4.0 International license.

### Motor intensity analysis.

The initial 30 frames of fluorescence images were linearly overlapped, and the peak pixel intensity was used to estimate the motor centroid, usually in agreement with that based on the center of rotation of the cell body in the corresponding bright-field images. Two regions of interest (ROIs) were defined centering on the motor centroid. The raw intensity of the motor (*I_raw_*) was calculated as the sum of all pixel intensities within the inner ROI of 7 × 7 pixels (455 × 455 nm). The background intensity (*B*) was defined as the mean pixel intensity within the outer ROI of 9 × 9 pixels (585 × 585 nm) but external to the inner ROI. However, the background intensity is not uniformly distributed throughout the cell due to rotation of the rod-shaped cell body (70). Therefore, the net motor intensity (*I*) should be calculated with the following equation:

(3) $$I\;=\;I_{\mathrm{raw}}-C\;\times \;B\;\times \;n$$

where *n* is the number of pixels within the inner ROI, and *C* is a correction factor for the estimation of background intensity in the motor region with the tethered-cell method under TIRF illumination. The mEGFP-producing strain XL2 carrying pAT5 (*pBAD33-megfp*) and pFD313 was used for estimating the correction factor *C* under experimental conditions identical to those used for motor intensity measurements (see Fig. S1A in the supplemental material). *C* was calculated as the ratio of *I_raw_* to (*B × n*). As shown in Fig. S1B, the correction factor *C* remains nearly constant (~1.1) before total photobleaching of mEGFP molecules. Therefore, the correction factor (*C*) was set to 1.1 for the calculation of motor intensity.

10.1128/mbio.00189-23.2 FIG S1 Correction of the background intensity. (A) Single frame of TIRF images from a tethered cell with uniformly distributed mEGFP molecules. Rotation of the rod-shaped cell causes a nonuniform distribution of fluorescence intensity. The outline of the cell is drawn with red dotted lines considering the orientation change due to the rotation in the exposure time of a single frame. The green round marker is the estimated motor centroid. The blue square represents the inner ROI of 7 × 7 pixels, and the yellow square represents the outer ROI of 9 × 9 pixels. (B) The correction factor (*C*) versus data acquisition time. Before total photobleaching of mEGFP molecules, *C* remained nearly constant with a value of ~1.1. Download FIG S1, TIF file, 2.0 MB. Copyright © 2023 Tao et al. 2023 Tao et al. https://creativecommons.org/licenses/by/4.0/ This content is distributed under the terms of the Creative Commons Attribution 4.0 International license.

### Quantitative Western blotting.

Cells were grown in 20 mL tryptone broth at 33°C to mid-log phase (OD_600_ = 0.6). A total of 15 mL of cell suspension was harvested by centrifugation, washed twice, and enriched in 1 mL of lysis buffer (25 mM Tris, 250 mM NaCl, 1 mM dithiothreitol [DTT], pH 7.5). Cell suspensions were lysed by sonication to prepare whole-cell extracts. Eighty microliters of whole-cell extracts was mixed with 20 μL of SDS-PAGE loading buffer (5×), heated to 100°C for 6 min, and cooled down to room temperature. Ten microliters of each sample mixture was loaded onto a 10% SDS gradient polyacrylamide gel. After separation by electrophoresis, the proteins were transferred to a 0.2 μm nitrocellulose membrane (Cytiva). The membrane was blocked with 5% (wt/vol) skim milk in Tris-buffered saline with Tween 20 (TBST) buffer (20 mM Tris, 150 mM NaCl, pH 7.4, 0.1% Tween 20) for 4 h at room temperature. Next, the membrane was incubated overnight at 4°C with 1:2,500 diluted monoclonal mouse anti-FliM antibody (Sangon Biotech) or 1:1,500 diluted monoclonal mouse anti-EGFP antibody (Abcam) in TBST containing 5% bull serum albumin and then incubated for 1 h at room temperature with 1:1,000 diluted horseradish peroxidase (HRP)-conjugated goat anti-mouse antibody (Invitrogen). Finally, the chemiluminescence signal was generated by an ECL detection kit (Thermo Scientific) and scanned with a chemiluminescence imaging system (Tanon).
