# Supplementary material for: Precise Measurement of the Stoichiometry of the Adaptive Bacterial Flagellar Switch
Source: mBio. 2023 Mar 22;14(2):e00189-23. doi: 10.1128/mbio.00189-23 (PMC10128058; doi:10.1128/mbio.00189-23)
Supplement: Text S1 [file mbio.00189-23-s0001.docx]

**Supporting Information**

**Comparison of the overall fluorescence efficiency of mEGFP fusions**

The present results are based on the prerequisite that different fusions of mEGFP molecules have comparable brightness. Even if we have considered the maturation time of mEGFP molecules in sample preparation, the chromophore of fluorescent protein (FP) cannot mature with 100% efficiency because of possible mistakes in chromophore folding, cyclization, oxidation, dehydration and others (1). The fraction of matured FPs is usually measured with biochemical assays such as quantitative immunoblotting (2). Since there are many characteristics associated with the fluorescence emission, such as the quantum efficiency and the fraction matured, that should be taken into consideration, we simplified the comparison by focusing on the overall fluorescence efficiency (*f*), which is defined as:

$$\text{f}\text{ }\text{ }\text{= }\frac{\text{F}}{\text{Q}_{\text{m}}} \text{,}\text{ } \text{[1]}$$

where *F* is the fluorescence emitted from a group of mEGFP molecules and *Q_m_* is the absolute quantity of mEGFP molecules. The intracellular fluorescence density (*D*) was calculated under the same induced levels as Western blot experiments for individual cells:

$$\text{D = }\frac{\text{F}_{\text{c}}}{\text{V}}\text{ = }\frac{\text{ }\text{f}\text{ }\text{·}\text{ }\text{C}\text{ }\text{·}\text{ }\text{V}}{\text{V}}\text{ }\text{=}\text{ }\text{f}\text{ }\text{·}\text{ }\text{C }\text{,}\text{ }\text{ } \text{[2]}$$

where *F_c_* is the net fluorescence emitted from the whole cell, *C* is the intracellular concentration of mEGFP molecules and *V* is the cell volume. The mean intracellular fluorescence density was further corrected by subtracting the background, that is, the mean intracellular fluorescence density of a non-fluorescent strain RP437. Therefore, the mean intracellular concentration of mEGFP molecules should be proportional to the mean intracellular fluorescence density in individual cells:

$$\text{ }\overline{\text{C}}\text{ = }\frac{\overline{\text{D}}}{\text{f}} . \text{ }\text{ }\text{ }\text{ }\text{ }\text{[3]}$$

For mEGFP-FliG induced by 130 μM IPTG and FliM-mEGFP induced by 100 μM IPTG, the results of Western blot experiments showed the ratio between the quantities of two kinds of fused mEGFP molecules contained in 15 mL cell culture. Moreover, we measured the dry weights of cells cultured to the same mid-log phase (OD_600_ = 0.6) as the Western blot experiments by lyophilization. We obtained a value of 0.16 ± 0.01 mg per mL of cell culture for both the mEGFP-FliG strain and the FliM-mEGFP strain. Details of this lyophilization procedure have been described in a previous study (3). The overall cell volume has been confirmed to be proportional to the dry weight (1.4 μL/mg) (4). We found that for individual cells, the cell volume (*V*) and intracellular fluorescence density (*D*) both showed a Gaussian distribution, and the correlation between *V* and *D* was negligible. Therefore, the mean intracellular concentration of mEGFP molecules could be approximated as (5):

$$\overline{\text{C}}\text{ ≈ }\frac{\text{Q}_{\text{wb}}}{\text{V}_{\text{wb}}}\text{ }\text{, [4]}$$

where *Q_wb_* is the overall quantity of mEGFP molecules measured by quantitative Western blot, and *V_wb_* is the corresponding cell volume calculated by the dry weight. By Eq. 3 and Eq. 4,

$$\frac{\overline{\text{D}}}{\text{f}}\text{ }\text{≈}\text{ }\frac{\text{Q}_{\text{wb}}}{\text{V}_{\text{wb}}}\text{ }\text{. }\text{ }\text{ }\text{ }\text{ [5]}$$

Since the dry weights of cell culture containing mEGFP-FliG and FliM-mEGFP are nearly the same in quantitative Western blot, the ratio of *f* is

$$\frac{\text{f}_{\text{M}}}{\text{f}_{\text{G}}}\text{ }\text{≈}\text{ }\frac{\text{Q}_{\text{w}\text{b}}^{\text{G}}}{\text{Q}_{\text{wb}}^{\text{M}}}\text{ · }\frac{\overline{\text{D}_{\text{M}}}}{\overline{\text{D}_{\text{G}}}}\text{ }\text{,}\text{ }\text{ }\text{ }\text{ }\text{[6]}$$

where the indexes “M” and “G” refer to FliM-mEGFP and mEGFP-FliG, respectively. The ratio of *f_M_* to *f_G_* is estimated to be 0.96 ± 0.24 according to the measured data. Therefore, the overall fluorescence efficiency is nearly the same in vivo regardless of the type of fusion.

**Detection of photons emitted from individual mEGFP molecules**

The strain HCB1357 (Δ*flhC*) expressing fused mEGFP molecules was used for single-molecule detection (see *Strains and plasmids*). This strain lacks the master regulator of flagellar transcription FlhC; thus, the cells are non-flagellated to avoid the aggregation of mEGFP molecules due to motor assembly (6). The expression of fused mEGFP molecules was induced to extremely low levels for single molecule detection. We used a Nikon N-STORM microscope with a highly sensitive EM-CCD (Andor DU897) to detect single-molecule fluorescence emission. The video was recorded under high-power TIRF illumination with an exposure time of 10 ms. In this case, if an mEGFP molecule drifted into the evanescent laser field, it was excited and recorded. Afterwards, individual emission bursts in the video were detected and localized by the Nikon NIS-Elements analysis software, which was based on a published method (7). Briefly, a two-dimensional Gaussian function was fitted to a 5 × 5 pixel block (800 × 800 nm) cropped around each local peak to obtain the centroid position, intensity, width, ellipticity and other characteristics of each burst. The peak intensity was converted to photon counts using the camera manufacturer’s calibrated curve for the electron multiplication and ADC gain settings used during imaging. Peaks too dim, too wide, or too skewed to yield satisfactory localization accuracy were abandoned for further analysis to reject some artifacts of bursts. As shown in Fig. S5, the highest local peak, which was also the first peak, corresponded to the photon count emitted from individual mEGFP molecules per frame. Under identical experimental conditions, all of the individual fused mEGFP molecules used in this study emitted the same number of photons regardless of the type of fusion, suggesting that the brightness of individual matured mEGFP molecules was comparable. Other peaks with photon counts about 2 or 3 times as large as the first peak also appeared, probably because two or three mEGFP molecules fortuitously drifted too close together to be distinguished.

**Supplemental references:**

1. Craggs TD (2009) Green fluorescent protein: structure, folding and chromophore maturation. *Chem. Soc. Rev.* 38(10):2865–2875.

2. Wang S, Moffitt JR, Dempsey GT, Xie XS, & Zhuang X (2014) Characterization and development of photoactivatable fluorescent proteins for single-molecule–based superresolution imaging. *Proc. Natl. Acad. Sci. U. S. A.* 111:8452–8457.

3. Tao A, Zhang R, & Yuan J (2020) Direct Mapping from Intracellular Chemotaxis Signaling to Single-Cell Swimming Behavior. *Biophys. J.* 119:2461–2468.

4. Stock JB, Rauch B, & Roseman S (1977) Periplasmic space in *Salmonella typhimurium* and *Escherichia coli*. *J. Biol. Chem.* 252(21):7850–7861.

5. Scharf BE, Fahrner KA, Turner L, & Berg HC (1998) Control of direction of flagellar rotation in bacterial chemotaxis. *Proc. Natl. Acad. Sci. U. S. A.* 95:201–206.

6. Liu X & Matsumura P (1994) The FlhD/FlhC complex, a transcriptional activator of the *Escherichia coli* flagellar class II operons. *J. Bacteriol.* 176:7345–7351.

7. Bates M, Huang B, Dempsey GT, & Zhuang X (2007) Multicolor super-resolution imaging with photo-switchable fluorescent probes. *Science* 317:1749–1753.
