# Supplementary figures and images for: Precise Measurement of the Stoichiometry of the Adaptive Bacterial Flagellar Switch
Source: mBio. 2023 Mar 22;14(2):e00189-23. doi: 10.1128/mbio.00189-23 (PMC10128058; doi:10.1128/mbio.00189-23)

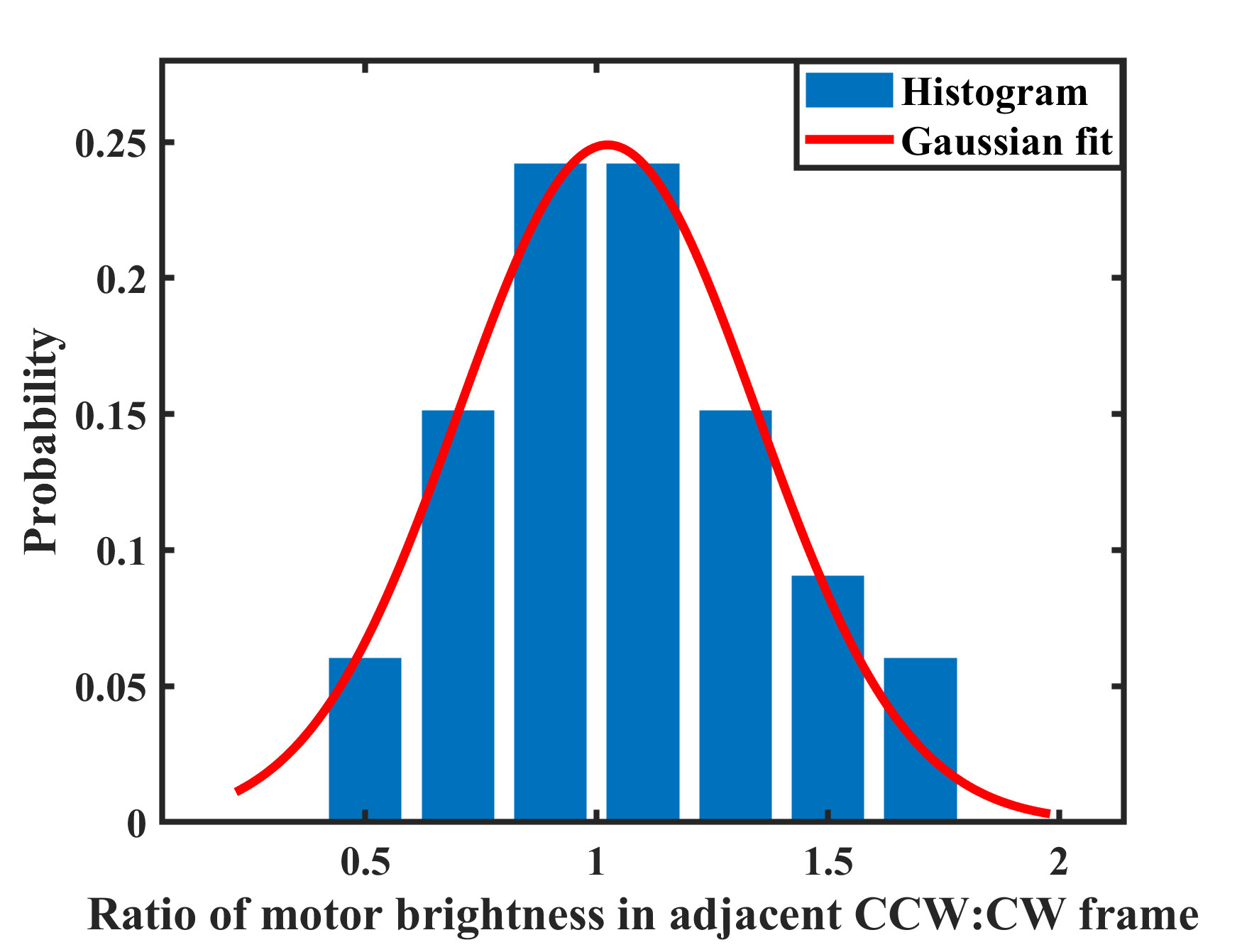

Supplement: FIG S3 [file mbio.00189-23-s0004.tif]

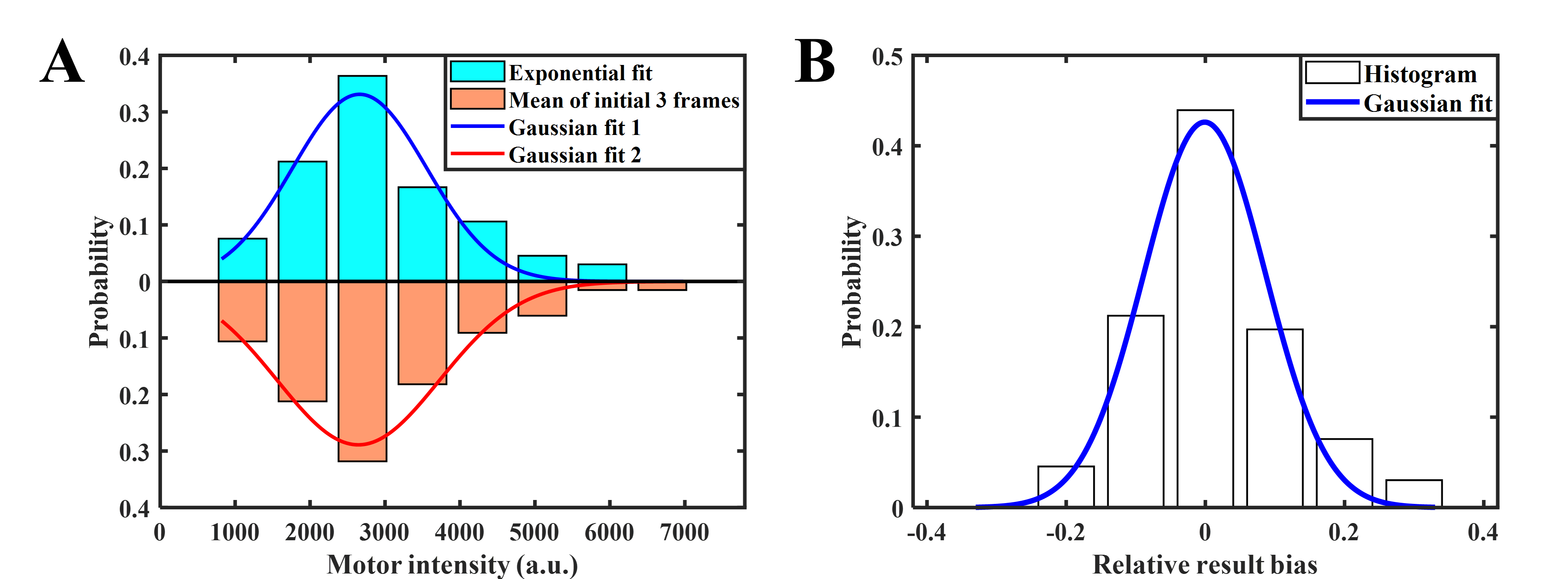

Supplement: FIG S4 [file mbio.00189-23-s0005.tif]

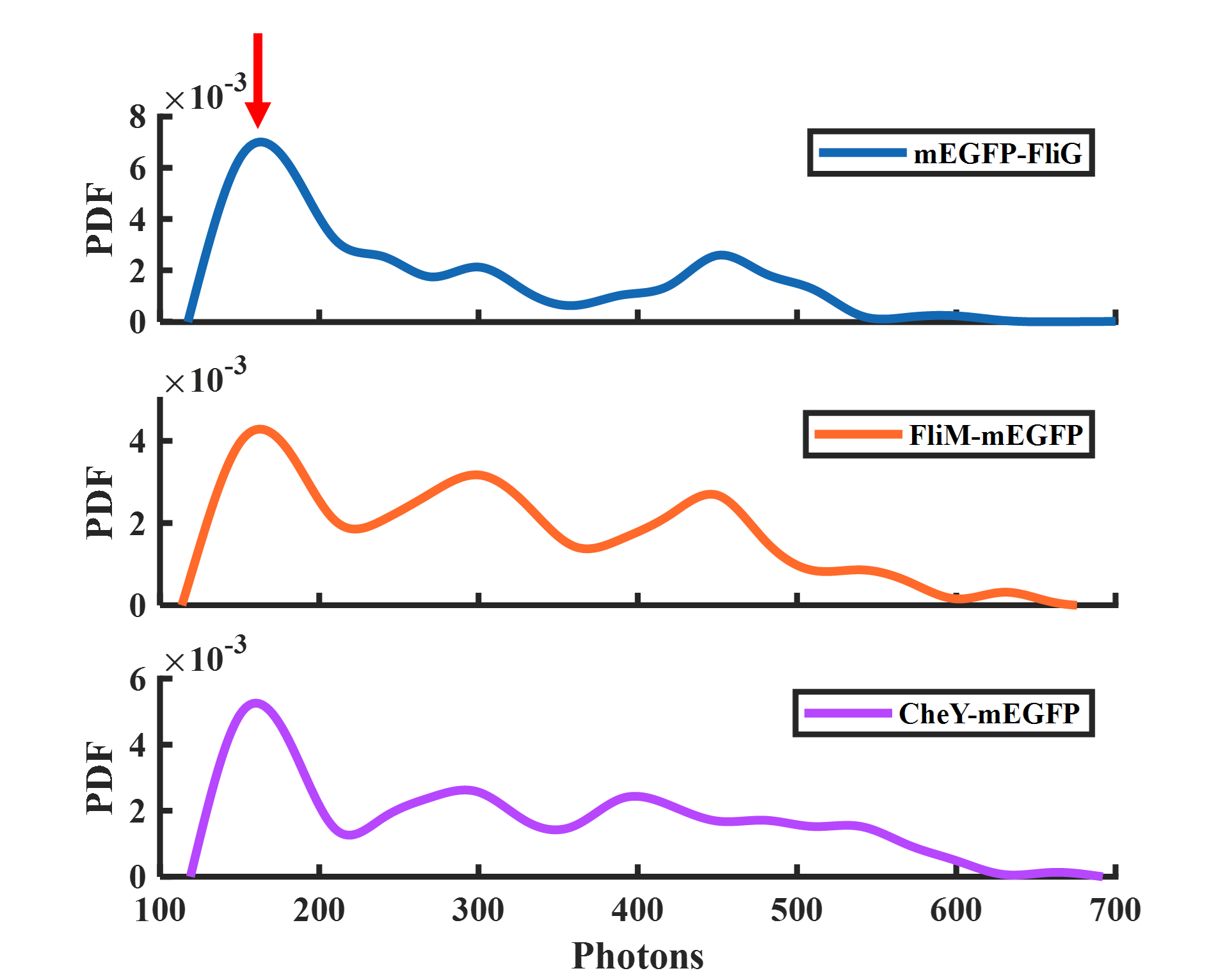

Supplement: FIG S5 [file mbio.00189-23-s0006.tif]

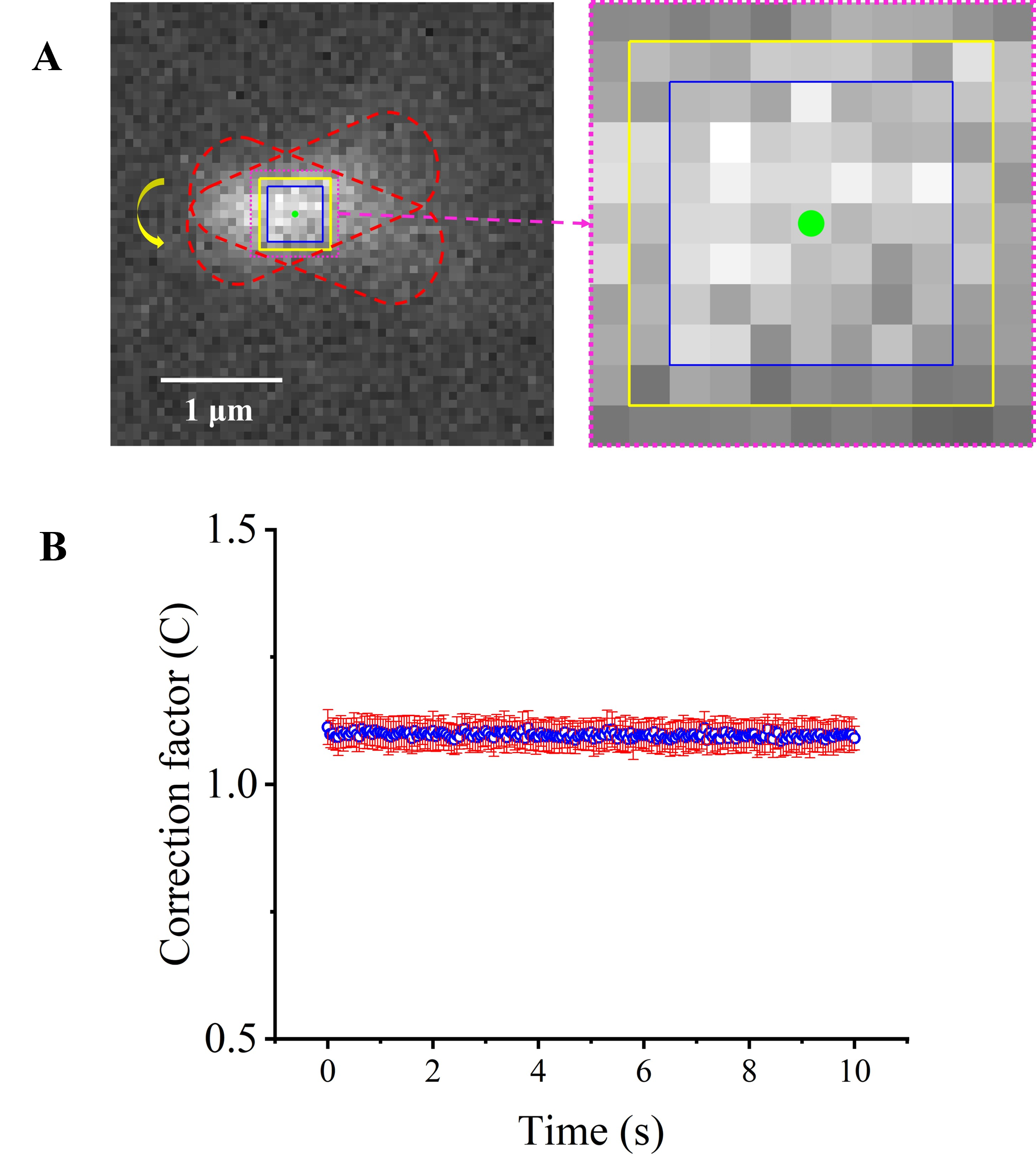

Supplement: FIG S1 [file mbio.00189-23-s0002.tif]
